# Ga(III) ion release from gallium-based liquid metals: The trade-off between efficacy and safety

**DOI:** 10.1016/j.isci.2026.117579

**Published:** 2026-09-21

**Authors:** Dawei Wang, Shuting He, Zhengkai Wei, Hong Tan, Yanni Wang, Junjie Lan, Weidong Pan

**Affiliations:** 1 Key Laboratory of Plant Resource Conservation and Germplasm Innovation in Mountainous Region (Ministry of Education), School of Pharmaceutical Sciences, Guizhou University, Guiyang, Guizhou 550025, China; 2 Guizhou Provincial Key Laboratory of Innovation and Manufacturing for Pharmaceuticals/Guizhou Engineering Laboratory for Synthetic Drugs, School of Pharmaceutical Sciences, Guizhou University, Guiyang, Guizhou 550025, China; 3 Department of Pharmacy, Guizhou Provincial People’s Hospital, Guiyang, Guizhou 550002, China; 4 State Key Laboratory of Discovery and Utilization of Functional Components in Traditional Chinese Medicine, Guizhou Medical University, Guiyang, Guizhou 561113, China

**Keywords:** gallium-based liquid metals, gallium-based LMs, gallium, Ga, trivalent gallium ions, Ga(III) ions, zero-valent gallium, Ga(0), efficacy, safety

## Abstract

Due to unique pharmacological activities and distinctive physicochemical properties, gallium-based liquid metals (LMs) have attracted tremendous attention in therapeutics. As biodegradable metallic materials, gallium-based LMs can predominantly release Ga(III) ions under physiological conditions, which represents a double-edged sword for clinical translation. On the one hand, the Ga(III) ions act as redox-inert Fe(III) competitive inhibitors and may provide therapeutic effects for broad-spectrum therapeutic applications. On the other hand, upon nonspecific biodistribution or uncontrolled Ga(III) release, substitution of Fe(III) by Ga(III) may disturb iron metabolism in normal cells and subsequently induce toxicological outcomes. Therefore, understanding and managing the trade-off between efficacy and safety is a key prerequisite for the clinical translation of gallium-based LMs. This review aims to systematically summarize the therapeutic activities and toxicological characteristics of gallium-based LMs. Meaningfully, the unresolved challenges and future perspectives regarding the balance between efficacy and safety are outlined to inspire future academic explorations and clinical translation.

## Graphical abstract

**Figure:**
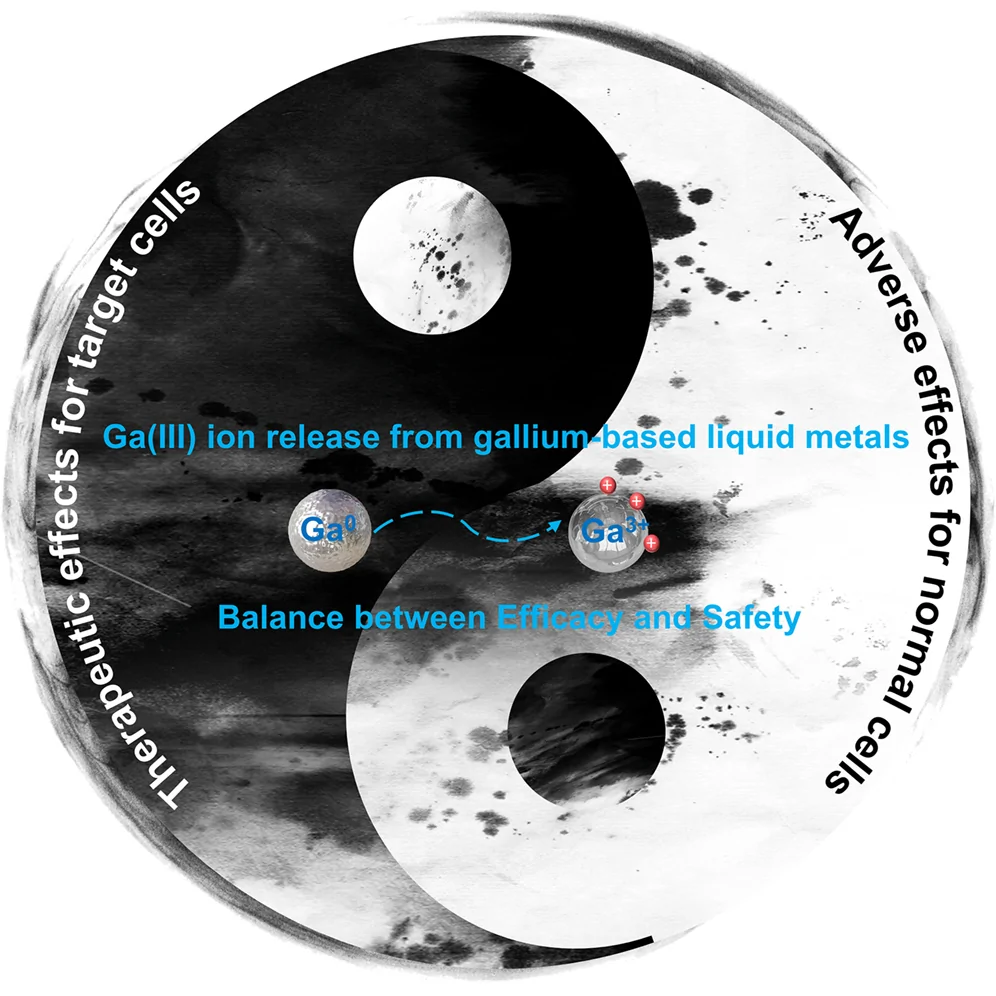

## Teaser

Chemistry; Engineering; Materials science

## Introduction

Metallic materials have become indispensable components in the modern biomedical field, with applications encompassing implantation, drug delivery, medical imaging, and tissue engineering, and so forth.^1^ From traditional implant substrates (e.g., stainless steel and titanium alloys) to functional metallodrugs (e.g., cisplatin and auranofin), research on biomedical metals continuously driven advances in both therapeutic technologies. Among diverse metallic biomaterials, gallium-based liquid metals (LMs, commonly defined as metals or alloys with melting points below or near room temperature^2^), typified by elemental gallium and gallium-indium alloys, have emerged as a highly promising category due to unique pharmacological activities and distinctive physicochemical properties.^3^^,^^4^^,^^5^^,^^6^^,^^7^ Despite the rapid expansion of research in this area, existing studies have largely focused on the functional performance of gallium-based LMs in specific biomedical applications, whereas comparatively less attention has been paid to their toxicological profiles and potential biological risks. Consequently, a critical knowledge gap remains regarding the systematic evaluation of efficacy and safety, which represents a foundation for promoting clinical translation.

As a multitarget pharmacologically active metallic element with long historical investigation, gallium in various forms (e.g., compounds, complexes, and so forth), has shown great potential in therapeutics,^8^ especially in antibacterial, anti-inflammatory, anticancer, osteogenesis, radio-pharmacology, molecular imaging and more emerging bio-related categories.^3^^,^^4^^,^^6^^,^^7^^,^^8^^,^^9^^,^^10^^,^^11^^,^^12^^,^^13^^,^^14^ Fundamentally, Ga(III), as a redox-inert Fe(III) competitive inhibitor, may interfere with iron metabolism, dysregulate cellular redox homeostasis, and modulate immune response, thereby exerting promising therapeutic activities.^6^^,^^11^^,^^14^^,^^15^ Recently, with physicochemical properties gradually elucidated, the gallium-based LMs have attracted increasing attention in the biomedical field. To date, diverse gallium-based LMs formulations, including nanomedicines, therapeutic sensitizers, drug carriers, bioactive materials, and biomedical devices, have been developed to facilitate precise treatment.^16^^,^^17^^,^^18^^,^^19^^,^^20^^,^^21^ These gallium-based LM formulations may not only act as sustained-release reservoirs for the active pharmaceutical ingredients (Ga(III)), but also serve as functional units to endow additional therapeutic activities. Typically, owing to their unique transformability and stimulus responsiveness, gallium-based LMs can exert therapeutic effects through heat accumulation, ROS generation, galvanic replacement, and catalytic effects.^5^^,^^14^^,^^22^^,^^23^^,^^24^^,^^25^^,^^26^^,^^27^ Meanwhile, they can be engineered as innovative responsive drug carriers for spatiotemporally controlled drug delivery.^5^^,^^6^^,^^23^^,^^25^^,^^28^^,^^29^^,^^30^^,^^31^^,^^32^ In addition, benefiting from radiopacity and electromagnetic properties, the gallium-based LMs can also be applied for X-ray/CT contrast enhancement,^33^^,^^34^ T2-weighted MRI contrast-negative enhancement,^35^ biomolecule detection,^36^^,^^37^ and photoacoustic (PA) imaging.^24^^,^^38^

The clinical translation of any biomedical material depends on its biosafety, biocompatibility, and toxicological characteristics, since these core prerequisites determine its safety and efficacy *in vivo*. With the growing attention devoted to gallium-based LMs, toxicological studies have emerged as an essential issue. Available clinical and preclinical evidence suggests that gallium exhibits relatively low cytotoxicity, hemotoxicity, hepatotoxicity, and histotoxicity when administered at appropriate doses.^6^^,^^14^^,^^34^^,^^39^ Although gallium is not an essential element for living organisms, it is widely considered to be biocompatible and non-toxic at low doses. This favorable safety profile is generally attributed to several factors: (1) It cannot enter red blood cells, thereby avoiding interference with oxygen transport; (2) It is biodegradable in physiological environments, for example, Ga^0^ may degrade or dissolve through redox reactions or oxidase reactions, releasing Ga(III) ions^40^; (3) it can be excreted via metabolic processes in the form of feces and urine.^5^^,^^7^^,^^41^ Collectively, the generally considered non-toxic and biodegradable characteristics lay the foundation for the safe applications of gallium-based LMs. However, considering that iron-dependent physiological processes (e.g., iron-catalyzed reactions, extracellular transport and cellular uptake^3^^,^^4^^,^^7^^,^^12^^,^^42^) are essential for all living organisms, the Ga(III) substitution strategy represents a double-edged sword (Scheme 1). On the one hand, Ga(III) can disrupt iron acquisition and utilization pathways in diverse biological systems, providing opportunities for broad-spectrum therapeutic interventions while reducing the likelihood of conventional resistance mechanisms.^3^^,^^4^^,^^6^^,^^12^^,^^14^^,^^43^^,^^44^ On the other hand, nonspecific biodistribution or unintended Ga(III) exposure may disrupt iron homeostasis in healthy tissues, thereby triggering a cascade of adverse biological responses and toxicological consequences.^4^

**Scheme 1:**
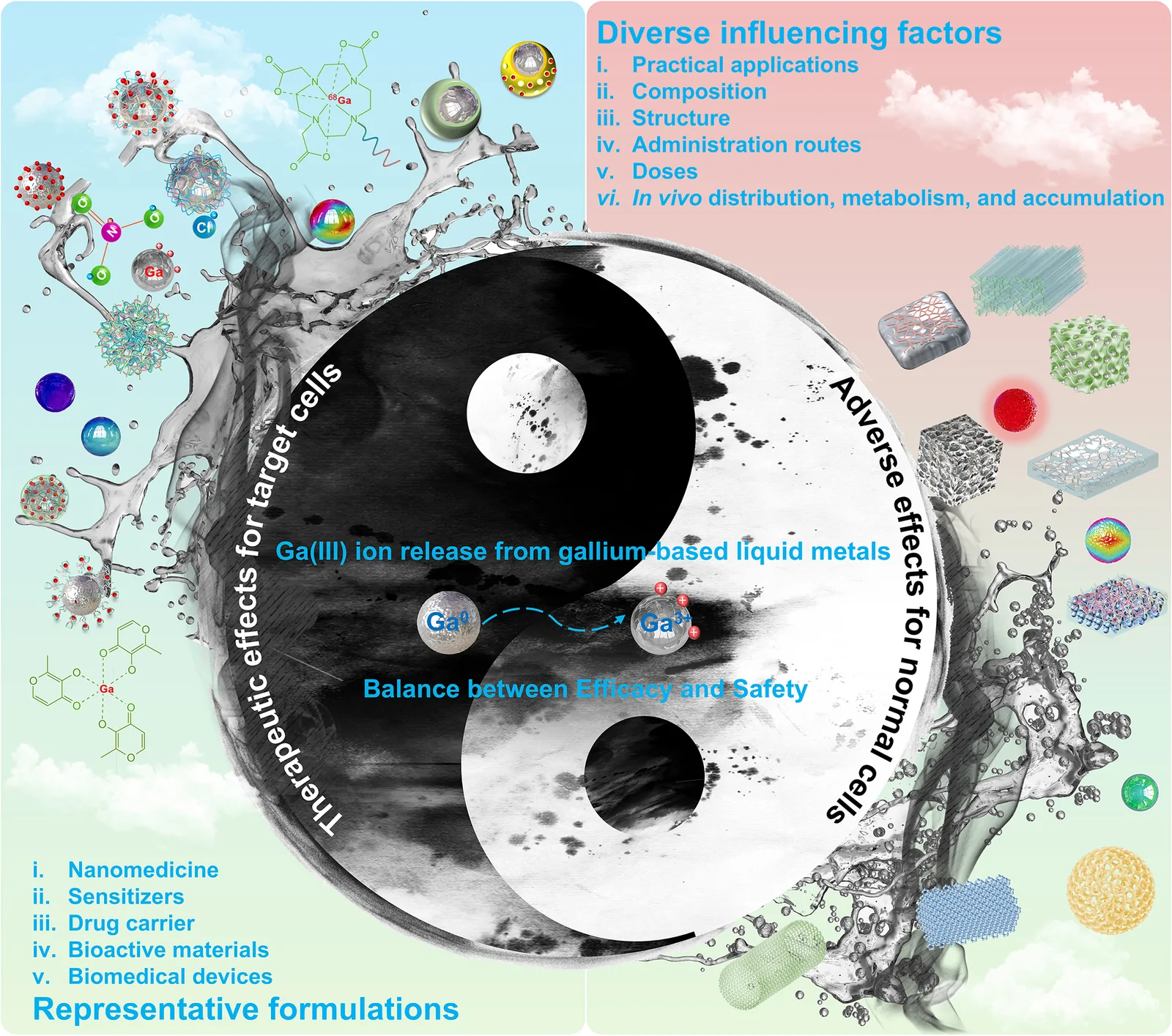
The Ga(III) ion release from gallium-based liquid metals represents a double-edged sword: therapeutic effects for target cells, adverse effects for normal cells

Up to now, reviews about gallium-based LMs in biomedical applications have emerged tremendously.^5^^,^^6^^,^^7^^,^^21^^,^^28^^,^^45^ However, few studies have specifically addressed the trade-off between efficacy and safety, nor the corresponding strategies for enhancing efficacy while reducing toxicity. Therefore, this review is dedicated to systematically identifying the therapeutic activity and toxicological characteristics of gallium-based LMs. The unresolved challenges and future perspectives for balancing efficacy and safety are also outlined to guide future investigations and accelerate clinical translation.

## Representative therapeutic activities of gallium-based LMs

As amorphous solids with fundamentally different mobility and metallicity,^2^^,^^14^ gallium-based liquid metals (LMs) possess a variety of inherent properties, e.g., high thermal and electrical conductivity, excellent fluidity, flexibility, and deformability, and other advantageous properties.^5^^,^^14^^,^^22^^,^^23^^,^^24^^,^^25^^,^^26^^,^^27^ Leveraging these properties, numerous biomedical applications have emerged in the field of therapeutics, while the underlying therapeutic activities and functionalities deserve particular attention.^5^^,^^6^^,^^23^^,^^25^^,^^28^^,^^29^^,^^30^^,^^31^^,^^32^

### The therapeutic activities via stimulus responsiveness

For therapeutic domains, the unique energy sensitivity of gallium-based LMs has garnered widespread attention.^2^^,^^46^ Gallium-based LMs are capable of interacting with external stimuli and the ambient environment, thereby converting exogenous energy into other forms (Figure 1A). Within the lesion, these stimulus-responsive properties hold great promise for multimodal therapeutic regimens, such as targeted thermotherapy/chemotherapy,^24^^,^^47^^,^^48^^,^^49^^,^^50^^,^^51^ dynamic therapy,^24^^,^^52^ mechanical therapy,^35^^,^^53^^,^^54^ and catalytic therapies.^55^^,^^56^^,^^57^^,^^58^

**Figure 1:**
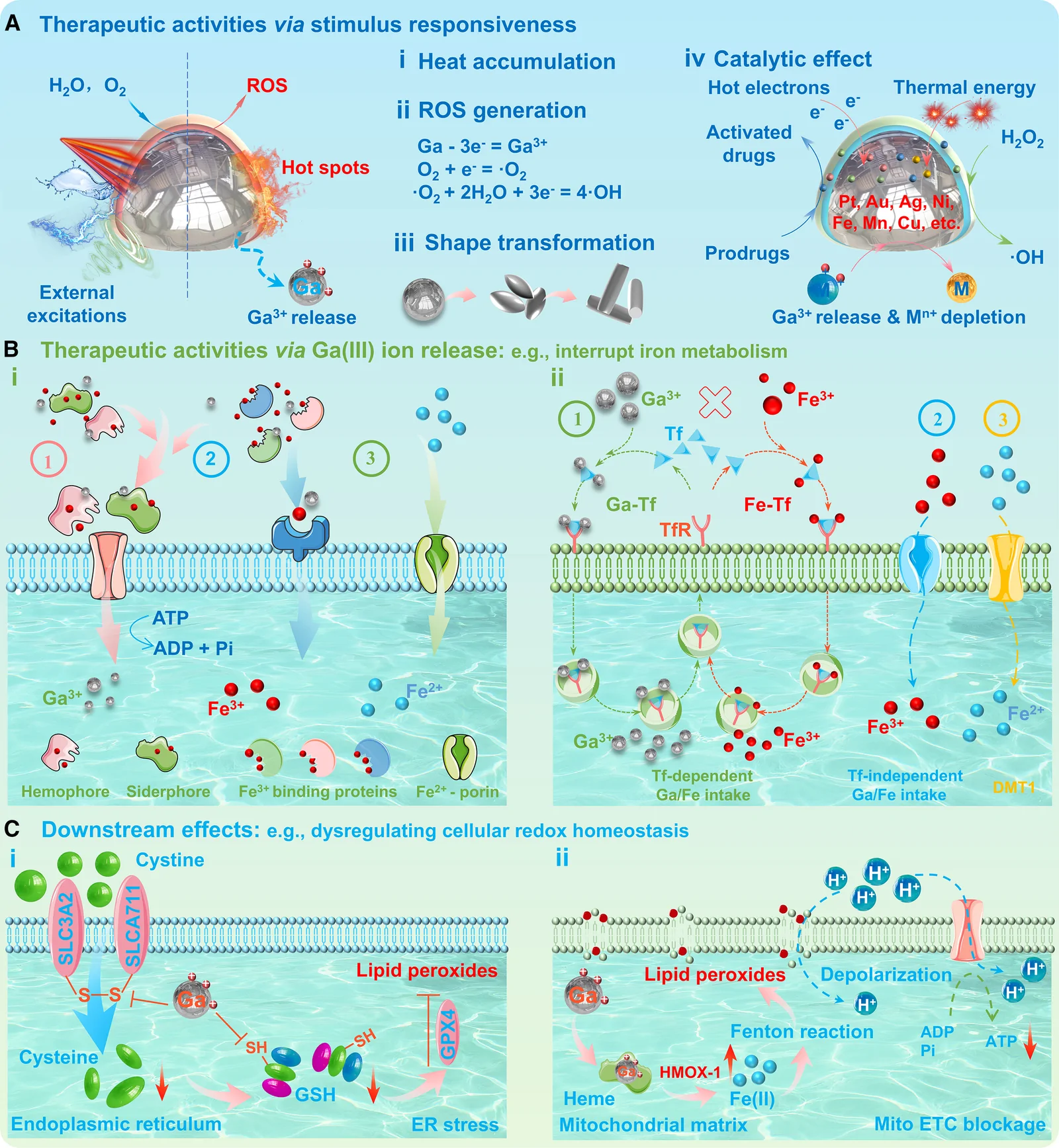
Representative therapeutic effects of gallium-based liquid metals (A) The stimulus responsiveness of gallium-based LMs, e.g., Ga(III) release, heat accumulation, ROS generation, shape transformation, and stimuli-enhanced catalytic effects. (B) Ga(III) as a non-functional Fe(III) mimic to interfere with iron metabolism, (i) prokaryotes (e.g., bacteria), (ii) eukaryotes (e.g., animals). (C) Downstream effects of Ga(III) substitution, e.g., dysregulation of cellular redox homeostasis (i: ER stress and enforced ferroptosis, ii: impaired membrane integrity), protein synthesis, enzyme activity, and gene expression. Created with Smart.Servier.com.

#### Thermal/dynamic response

Among these functionalities, stimulus-induced thermal conversion is the most extensively investigated. For example, under light irradiation at specific wavelengths, the gallium-based LMs may exhibit localized surface plasmon resonance (LSPR) effect,^59^^,^^60^ resulting in efficient photothermal conversion with reported efficiencies ranging from 25.3% to 53.0%.^46^ While the similar thermal response phenomena have also been observed under other external stimuli, including magnetic, electrical, and acoustic stimulation.^46^ For example, due to favorable electrical conductivity, the gallium-based LMs also exhibit a remarkable magnetocaloric phenomenon under alternating magnetic fields (AMF).^47^^,^^48^^,^^49^^,^^50^^,^^51^ Benefiting from the electrochemical reactions triggered by thermal energy and the ambient environment, the gallium-based LMs may also generate ROS and undergo sharp physical deformations.^24^ Specifically, the ROS generation is propelled by electron transfer from Ga(0) to oxygen and water (Ga - 3e = Ga^3+^, O_2_ + e = ·O_2_, ·O_2_ + 2H_2_O + 3e = 4·OH), while the stimuli-triggered deformation is caused by the heat-induced oxidation (intermediate crystal product, GaOOH: 4Ga +3 O_2_ + 2H_2_O → 4GaOOH, 2Ga + 2H_2_O → 2GaOOH + 3H_2_ ↑) and fluidic nature.

#### Catalytic effect

In addition, the gallium-based LMs may also facilitate *in vivo* catalytic reactions, thereby enabling externally stimulated therapeutic activities. Owing to the fluidic nature and electron-rich environment, numerous studies have demonstrated the potential of gallium-based LMs for constructing highly efficient liquid catalytic systems, particularly when doped with additional trace metallic elements (e.g., Au, Pt, Ag, Ni, Cu, and so forth).^55^^,^^56^^,^^57^^,^^61^^,^^62^^,^^63^^,^^64^^,^^65^^,^^66^ Under physiological conditions, these gallium-based LM catalyst systems are capable of initiating catalytic reactions, e.g., bioorthogonal catalysis and Fenton-like catalysis, thereby activating prodrugs and generating ROS.^55^^,^^56^ Meanwhile, the thermal energy and hot electrons triggered by external stimuli (e.g., light, ultrasound, and so forth) will further enhance catalytic efficiency and amplify therapeutic efficacy.^55^^,^^56^^,^^57^^,^^58^

#### Flexible and active drug transport

Unlike conventional “static” or “rigid” metals, gallium-based LMs possess distinctive transformability, degradability, and stimulus responsiveness. Thus, the gallium-based LMs exhibit remarkable advantages in flexible and active drug transport.^9^ For example, the gallium-based LMs with adaptive deformability may enable drug transport and accumulation via intracellular/intercellular routes; While the mildly acidic endosomal/lysosomal microenvironments will promote their fusion and degradation, thereby enabling slowly and sustained release of loaded drug and Ga(III) ions.^15^^,^^67^ Notably, the controlled actuation of gallium-based LM carriers under external stimuli may endow them with active targeting capabilities.^67^^,^^68^^,^^69^ Moreover, the external stimuli-induced physical/chemical changes can also be utilized to promote drug release from gallium-based LMs carriers (for details, see Wang et al.^9^). Examples include mutual fusion-induced drug release, thermal-induced drug release, ion exchange-enabled drug release, and deformation-induced drug leakage, which constitute effective strategies for achieving spatiotemporally controlled drug delivery.^34^^,^^39^^,^^47^^,^^48^^,^^67^^,^^68^^,^^70^

### The therapeutic activities via Ga(III) ion release

Gallium ions (Ga(III)), as multitargeted pharmacologically active metallic ions, have demonstrated considerable potential in antibacterial, antineoplastic, anti-inflammatory, osteogenesis, and more emerging categories.^6^^,^^11^^,^^14^^,^^15^ Owing to the analogous chemical behaviors, Ga(III) tends to compete with iron ions (Fe(III)), thereby interfering with iron metabolism and inducing downstream biological responses.^42^ This Fe(III)-mimicking behavior is widely recognized as the principal mechanism underlying the therapeutic activities of gallium-based systems.^7^

#### Ga(III) release

Since gallium exhibits amphoteric chemical properties, an atomic oxide layer (Ga_2_O_3_, 0.7–3 nm^71^^,^^72^) may form at the interface between gallium-based LMs and the surrounding environment.^14^^,^^73^ This surface oxide layer exhibits partial passivation similar to aluminum oxide, but may be reversed when exposed to external excitations (e.g., chemical, electrical, mechanical, pressure, and temperature, and so forth).^14^ For instance, under specific pathological conditions (such as a mildly acidic environment within tumors, infected wounds and endosomal/lysosomal microenvironments), this oxide layer may dissolve, thereby exposing pristine gallium-based LMs. More importantly, among the main components of the gallium-based LMs, Ga (−0.56 V) possesses lower reduction potential (E^0^) than In (−0.34 V), and Sn (−0.14 V), hence metallic gallium (Ga(0)) will undergo redox reactions preferentially. Therefore, the gallium-based LMs can serve as a reservoir to dominantly release Ga(III) ions,^40^ which constitutes a critical foundation for therapeutic applications (Figure 1A). Notably, leveraging the sustained-release properties of Ga(III), gallium-based LMs can be utilized to simplify therapeutic strategies and enhance the efficacy of targeted therapy.^9^ For example, the surface-modified EGaIn nanodroplets can be administered orally to treat inflammatory bowel disease, while achieving enhanced bioavailability and reduced systemic adverse effects.^20^ The release of Ga(III) from gallium-based LMs is typically accompanied by electrochemical reactions, which are expected to confer additional therapeutic activity via disruption of ion balance and intracellular redox homeostasis.^14^^,^^19^^,^^20^ Given the electrochemical potential window of Ga(0), i.e., E^0^: Zn (−0.763V) < Ga (−0.56 V) < Fe (−0.41 V) < Cu (+0.34 V), the gallium-based LMs are capable of modulating endogenous metal concentrations through galvanic replacement reactions. Taking copper (Cu) depletion therapy as an example, the gallium-based LMs nanodroplets may efficiently consume intracellular Cu ions (electrochemical substitution, E^0^: Ga (−0.56 V) < Cu (+0.34 V)), while simultaneously facilitates the Ga(III) release and reactive oxygen species (ROS) generation (Figure 1A).^19^ This *in vivo* electrochemical substitution strategy may offer remarkable therapeutic promise for versatile biological systems, whereas it also raises concerns regarding biosafety.

#### Interfere with iron metabolism

In prevalent cationic state, Ga(III) and Fe(III) share remarkable physicochemical similarities,^6^^,^^74^ especially in ionization energies (Ga(III): 64 eV, high-spin Fe(III): 54.8 eV), electron attractions (Ga(III): 30.71 eV, akin to high-spin Fe(III): 30.65 eV), octahedral and tetrahedral ionic radii dimensions (Ga(III): 0.62 Å and 0.47 Å, high-spin Fe(III): 0.645 Å and 0.49 Å). However, there is only one stable oxidation state for gallium,^75^ but iron may switch between trivalent Fe(III) and divalent Fe(II). Therefore, Ga(III) is considered to be a redox-inert Fe(III) competitive inhibitor. It is worth mentioning that iron-dependent physiological processes are essential for the survival and proliferation of various types of living organisms, including healthy cells, pathogens (e.g., bacteria), and malignant cells (e.g., cancer cells).^74^ Since pathogens and malignant cells are characterized by rapid metabolism and proliferation, they require more iron to combat iron deficiency.^76^ While the major strategies for iron acquisition are summarized in Figure 1B. In bacteria, iron acquisition primarily occurs through three mechanisms: (1) secret various ferric complexes (e.g., ferric chelating hemophores and siderophores) for active intracellular Fe(III) transport^77^; (2) acquire Fe(III) from iron-binding proteins via specific transport systems (e.g., transferrin (Tf))^78^; and (3) directly uptake Fe(II) via the ferrous iron (Feo) transport systems.^79^ In cancer cells, iron uptake pathways include (1) Tf and transferrin receptor (TfR) mediated pathway, (2) Tf-independent pathway, and (3) divalent metal transporter 1 (DMT1).^7^ In this regard, gallium as a novel candidate for iron-blocking antibacterial and antineoplastic therapies, may effectively inhibit the growth of common pathogenic bacteria (e.g., *E. coli*, *P. aeruginosa*, and *S. aureus*) and various malignancies (e.g., breast cancer, lung cancer, and liver cancer).^75^ Importantly, these iron-blocking therapies are less susceptible to drug resistance caused by factors such as target mutations, altered metabolic processes, signaling pathway reorganization, drug effect diminution, and so forth.^43^^,^^44^

#### Downstream biological effects

Beyond disrupting iron acquisition, Ga(III) may further perturb multiple cellular processes, including redox homeostasis, protein synthesis, enzyme activity, and gene expression, thereby giving rise to diverse downstream therapeutic effects. For example, studies have demonstrated that Ga(III) in the form of compounds or complexes can regulate the expression of metallothionein and heme oxygenase-1 (HMOX-1) through mitochondrial ROS generation.^80^^,^^81^ Subsequently, the pioneering studies have confirmed that Ga(III) may induce ferroptosis through cellular redox homeostasis dysregulation.^82^^,^^83^ For instance, the Ga(III) complex capable of targeting protein disulfide isomerase (PDI) was shown to i) prevent the repair of the antioxidant glutathione (GSH) system and ii) disrupt membrane integrity to trigger intracellular iron overload. These events ultimately promote lipid peroxidation, endoplasmic reticulum (ER) stress, and ferroptosis (Figure 1C).^13^^,^^84^ Besides, the Ga(III) substitution may disrupt iron homeostasis and further impede the native protein functions in immune cells, thus exerting anti-inflammatory activity.^3^^,^^4^^,^^85^ As a source of Ga(III) ions, gallium compounds have historically been utilized as anti-inflammatory agents for treatment of autoimmune encephalomyelitis,^86^ inflammatory arthritis,^87^ and systemic lupus erythematosus.^88^ While the mechanistic studies have shown that the anti-inflammatory effects stem from the regulation of pro-inflammatory cytokines and nitric oxide (NO) produced by activated immune cells.^87^^,^^89^^,^^90^ In addition, the Ga(III) released from gallium-based LMs can also indue multiple biological effects, including activation of pattern recognition receptors (e.g., TLR, NLR, and STING), triggering of the NF-κB inflammatory pathway, promoting cytokines (e.g., IFN-I and IL-1β) secretion, upregulating major histocompatibility complex (MHC) expression, and other physicochemical interactions (e.g., ion imbalance, lysosomal disruption, and mitochondrial stress). Based on these biological effects, recent studies have demonstrated the immunostimulatory capabilities of gallium-based LMs, which can enhance antigen cross-presentation to promote dendritic cells maturation/activation, thereby driving cytotoxic T lymphocytes memory for cancer immunotherapy.^15^^,^^91^^,^^92^^,^^93^^,^^94^^,^^95^ Moreover, the Ga(III) may also exert dual role in inhibiting bone resorption (anti-osteoclastic effect) and promoting bone regeneration (osteogenic activity), which hold great potential for the treatment of a variety of bone-related diseases (e.g., osteoporosis, bone destructive diseases, and dental/orthopedic implants).^7^^,^^45^^,^^96^^,^^97^^,^^98^^,^^99^^,^^100^^,^^101^^,^^102^^,^^103^^,^^104^ The anti-osteoclastic effects are attributed to the regulation of specific osteoclast differentiation early markers (e.g., NFATc1, FRA-2, JDP-2 and JUND), inhibition of enzymes secretion involved in bone matrix degradation and resorption, and modulation of calcium homeostasis.^105^^,^^106^^,^^107^ In parallel, the Ga(III) preferentially accumulates in active bone-forming regions,^108^ may further enhance the early differentiation of the osteoblast phenotype to promote bone regeneration.^97^^,^^104^

Overall, gallium-based LMs and released Ga(III) ions exhibit considerable therapeutic potential across a wide range of biomedical applications. However, the disruption of critical cellular processes and the associated downstream biological effects may also lead to unintended toxicological consequences, highlighting the importance of carefully balancing therapeutic efficacy with biosafety.

## Toxicological characteristics of GA (III) based formulations

Under physiological conditions, gallium-based LMs may gradually degrade and release Ga(III) ions, representing the primary form involved in biological processes and potential toxicological reactions. Thus, understanding the toxicological characteristics and possible hazards of Ga(III) ions is critical for assessing the biosafety of gallium-based LMs. Fortunately, abundant preclinical and clinical investigations of Ga(III) compounds and complexes have defined the toxic doses of Ga(III) ions, which may provide fundamental references for toxicological evaluation of gallium-based LMs. To provide a quick overview of the toxic doses, the administration routes, *in vivo* dosage, dosing frequency, and safe dosage are summarized in Table 1.

**Table 1:** Toxicological characteristics of Ga (III) based formulations, derived from clinical and preclinical studies

Classification	Abbreviation	Administration routes	Pharmacological activities	Application scenarios	*In vivo* dosage	Dosing frequency	Safe dosage	Year	Reference
Ga(III) compounds	gallium nitrate	intravenous administration	inhibiting bone resorption	cancer-related hypercalcemia	Ga: ∼1.47 mg/kg/d	5 consecutive days	plasma drug concentration: Ga ∼1.1–2.4 μg/mL; Discontinue immediately if serum creatinine >2.5 mg/dL	1990	Hall and Chambers^101^
		intraperitoneal administration	antitumor	subcutaneous solid tumor	*LD*_*50*_: Ga ∼18.40 mg/kg/d for rats; ∼21.81 mg/kg/d for mice	10 consecutive days	nephrotoxicity observed in all test subjects	1971	Hart et al.^109^
		oral administration	none	none	LD_50_: Ga ∼480 mg/kg for rats, 590 mg/kg for mice	Single dose	*LD*_*50*_/(100–1000)	1987	Domingo et al.^110^
	gallium sulfate	oral administration	none	none	LD_50_: Ga ∼330 mg/kg for rats, 380 mg/kg for mice	Single dose	*LD*_*50*_/(100–1000)	1987	Domingo et al.^110^
	gallium chloride	oral administration	antitumor	lung cancer	Ga: 0.66–5.26 mg/kg/d	10 consecutive days	optimal dosage: Ga: ∼2.64 mg/kg/d	1989	Collery et al.^111^
Ga (III) complexes	gallium maltolate	Oral administration	antitumor	lymphoma	Ga: 0.262–1.31 mg/kg	Single dose	no serious adverse effects	2000	Chitambar et al.^112^
		Peritumoral injection	antitumor	human cutaneous t cell lymphoma tumor	Ga: ∼3.13 mg/kg/d	5 consecutive days	no serious adverse effects	2014	Bernstein et al.^113^
	KP46	Oral administration	antitumor	malignant solid tumors	Ga: ∼0.11–1.80 mg/kg/d	14 consecutive days	no visible toxicity	2009	Timerbaev^114^

### Ga(III) compounds

Owing to weak coordination capacity, gallium compounds, e.g., gallium nitrate (Ga(NO_3_)_3_), gallium sulfate (Ga_2_(SO_4_)_3_), gallium chloride (GaCl_3_), may completely ionize to release Ga(III) ions. Accordingly, toxicological evaluations of these compounds offer a more accurate representation. Following single oral administration of gallium nitrate, the median lethal dose (LD_50_) was Ga = 480 mg/kg for rats and 590 mg/kg for mice. For gallium sulfate administered via the same route, the corresponding LD_50_ values were Ga = 330 mg/kg for rats and 380 mg/kg for mice (Table 1). However, the adverse effects, such as anorexia, weight loss, diminished pupillary reflexes, exophthalmos, muscle atrophy, and so forth, were observed during the 14-day observation period.^11^ Thus, the toxicities of gallium compounds exhibit clear dose and time dependence. Notably, Ga(III) compounds may undergo hydrolysis (e.g., at a physiological pH ∼7.4, Ga(OH)_3_ and Ga(OH)_4_^-^ hydroxides^115^), which not only significantly impairs the bioavailability and pharmacological efficacy, but also affects the assessment of toxicity.^9^^,^^13^ In fact, the low toxicity of orally administered Ga(III) compounds is likely attributable to their reduced bioavailability. In contrast, both acute and chronic toxicity is more pronounced following injection, e.g., intraperitoneal, subcutaneous, intramuscular, and intravenous injection. Generally speaking, relevant acute toxicology data indicate that injection^13^ of gallium-based substances is far more toxic than oral administration.^18^^,^^20^^,^^116^ For example, intraperitoneal administration of gallium nitrate to rats and mice for 10 consecutive days resulted in renal toxicity, with LD_50_ values of 67.5 mg/kg/d and 80 mg/kg/d, respectively (Table 1).^109^ Based on clinical pharmacokinetic studies, the safe doses are more stringent when administered intravenously. For gallium nitrate, the first FDA-approved gallium-based drug, the therapeutic plasma concentration of Ga should be maintained at 1.1–2.4 μg/mL when treating cancer-related hypercalcemia,^101^ while the therapy should be discontinued if the serum creatinine level exceeds 2.5 mg/dL (Table 1, U.S. FDA. Ganite®, NDA 019961/S-009, 2003.).

### Ga(III) complexes

As 2^nd^ generation Ga(III) therapeutic agents, the Ga(III) complexes may prevent hydrolysis through chelation of organic ligands, thereby largely overcoming the unfavorable pharmacokinetic and toxicological characteristics of gallium compounds.^13^^,^^114^ As prime examples, gallium maltolate (GaM, tris(3-hydroxy-2-methyl-4*H*-pyran-4-onato)gallium^113^^,^^117^) and tris(8-quinolinolato)gallium(III) (KP46)^114^ are the most representative candidates. Specifically, the antineoplastic activity of GaM and KP46 is greatly enhanced against different cell lines (IC_50_ 10–40 μM for GaM,^112^ and 1–3 μM for KP46,^114^ respectively. Unlike gallium compounds, gallium complexes possess better kinetic stability and gastrointestinal absorption, allowing for low-dose oral administration with greatly improved biosafety.^118^ For specific Ga(III) complexes, there may also be differences in cellular uptake, molecular interactions and biological effects, thus their biological behaviors cannot simply be equated with that of Ga(III) compounds.^13^ According to the clinical trial results, healthy human subjects showed excellent tolerability to single doses of GaM (100, 200, 300, and 500 mg, containing 15.7, 31.3, 47.0, and 78.4 mg of Ga), with no serious adverse events reported (Table 1).^113^ For patients with advanced malignant solid tumors (ovarian, renal, gastric, and parotid gland tumors), KP46 orally administered at doses from 30 to 480 mg/m^2^/d over two weeks was also very well tolerated with no dose-limiting toxicities observed (Table 1).^114^

## Toxicological characteristics of gallium-based liquid metals

### Toxicological characteristics of core elements

The toxicity of gallium-based LMs essentially depends on the toxicological characteristics of core elements, such as gallium (Ga), indium (In), and tin (Sn). As the most extensively studied component, Ga is widely considered to be biocompatible and non-toxic at low doses, although it is not an essential element for living organisms. Compared with other low melting point (below 40°C) metallic elements, e.g., mercury (Hg), rubidium (Rb), cesium (Cs), and francium (Fr), Ga exhibits superior biocompatibility with a tolerated dose of up to 500 mg in healthy individuals.^2^^,^^5^^,^^8^^,^^9^ The main reasons can be attributed to: (1) intrinsic features of non-radioactive (^69^Ga) and low vapor pressure (∼10^−35^ Pa at 30°C); (2) inability to enter erythrocytes, thus avoiding interference with oxygen transportation; (3) biodegradability under physiological conditions^40^; and (4) efficient excretion through feces and urine.^5^^,^^7^^,^^41^ Therefore, these toxicological characteristics lay the foundation for the safe biomedical applications of gallium-based LMs. However, high-dose exposure of In and Sn should be avoided, since In is slightly radioactive while excessive ingestion of Sn may cause adverse effects (e.g., neurotoxicity).^41^

### Toxicological characteristics of representative formulations

The toxicological behavior of gallium-based LMs is influenced by multiple variables, including formulation, administration route, dose, application scenarios, species, and sex differences, and so forth.^5^^,^^6^^,^^13^^,^^21^^,^^45^ Among these variables, the formulation is particularly important since it determines the administration route, dose, therapeutic efficacy, and intended applications. The typical formulations of gallium-based LMs for therapeutic domains are summarized in Figure 2, including nanomedicines, sensitizers, drug carriers, bioactive materials, and biomedical devices, and so forth. The corresponding administration routes, pharmacological activities, application scenarios, *in vitro* and *in vivo* dosages are illustrated in Table 2.

**Figure 2:**
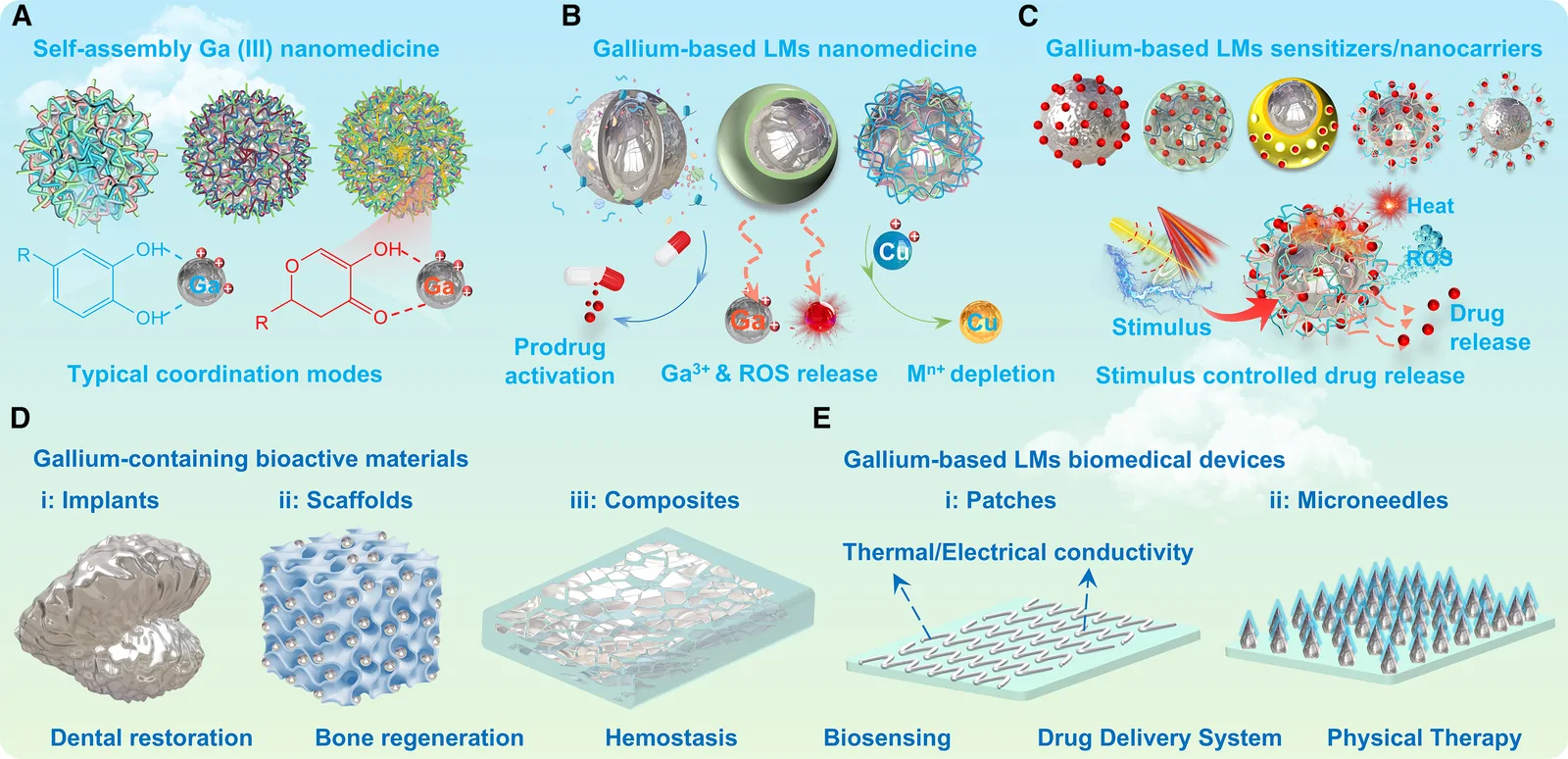
Representative formulations (A) Ga (III) based nanomedicines via self-assembly of Ga(III) ions with ligands. (B) Gallium-based LMs nanomedicine utilizing the intrinsic catalytic effect and released Ga(III) ions. (C) Gallium-based LMs for energy sensitizers and drug carriers. (D) Ga-containing bioactive materials, e.g., dental/orthopedic implants, bone regeneration scaffolds, hemostatic composites. (E) Gallium-based LMs for biomedical devices, e.g., biosensing devices, transdermal patch, and microneedles.

**Table 2:** Administered dosage of representative formulations, derived from current studies

Classification	Abbreviation	Other substance added/formed (and their purpose/role)	Administration routes	Size	Pharmacological activities	Application scenarios	*In vitro* dosage	*In vivo* dosage	Year	Reference
Gallium-based LMs nanomedicine	LMNDs	DOX (stabilization)	intratumoral administration	∼20 nm	antitumor	breast cancer (BCap-37 cells)	IC_50_ = 30.4 μg/mL	5 mg/kg	2023	Yan et al.^19^
	LM-EGCG	mPEG-SH (stabilization)	oral administration	∼250 nm	antibacterial; anti-inflammatory	inflammatory bowel disease (Escherichia_Shigella)	Ga: ∼100 μg/mL	Ga: ∼5 mg/kg	2024	Liu et al.^20^
	DPMG	DSPE-PEG2000-Mal (enhances antigen adsorption)	intratumoral administration	∼130 nm	antitumor	breast cancer (4T1 cells)	Ga: ∼1 mg/mL	Ga: ∼2.5 mg/kg	2023	Wang et al.^15^
Gallium-based LMs sensitizers	LM@GOX	mPEG-SH (stabilization); GOX: (catalysis)	intravenous administration	98.21 nm	antitumor	breast cancer (4T1 cells)	Ga: ∼4.72 μg/mL	Ga: ∼11.33 mg/kg	2019	Hu et al.^119^
	LM@MSN/DOX@HA	HA (targeting); DOX (antitumor); MSN (delivery carrier)	intravenous administration	160.4 nm	antitumor	breast cancer (4T1 cells)	Ga: ∼6 μg/mL	Ga: ∼0.75 mg/kg	2019	Hu et al.^70^
	GLM-Fe	Fe (magnetic iron particle)	none (*in vitro*)	∼200 nm to ∼2 μm	antibacterial	*P. aeruginosa*, *S. aureus*	100 μg/mL	None	2020	Elbourne et al.^120^
	PEG-IL-LM-ZrO_2_ SNPs	PEG (stabilization); IL (MW-sensitive effect)	tail vein injection	210 ± 60 nm	antitumor; CT imaging	liver cancer (H22 tumor cells, Hepa 1–6 tumor cells)	100 μg/mL	50, 100, 150 mg/kg	2019	Wu et al.^52^
	Ga/MPs	C8161 cell membrane (targeting); Paclitaxel (antitumor)	intratumoral injection	1 μm	antitumor; CT imaging	skin cancer (C8161 cells)	400 μg/mL	500 mg/kg	2022	Wang et al.^54^
	LM-Pd	LM (modulating biorthogonal catalysis); Pd (catalyst)	intravenous administration	∼210 nm	antitumor	colon cancer (CT26 cells)	300 μg/mL	16 mg/kg	2023	Zhang et al.^55^
Gallium-based LMs drug carrier	LMGNS	Leukocyte Membrane (active targeting); DOX·HCl (antitumor)	none (*in vitro*)	∼7 μm in length; diameters of ∼800 nm and ∼150 nm at each end (needle-like shape)	antitumor	Cervical cancer (HeLa cells)	10 μg/mL	None	2020	Wang et al.^68^
	tNPs	Graphene quantum dots (enhance functionality) and DOX (antitumor)	intravenous injection	∼100 nm	antitumor	Cervical cancer	Dox: IC_50_ = 0.35 mg/L	Maximum tolerated dose: 750 mg/kg	2017	Lu et al.^69^
Gallium-containing bioactive materials	EGaIn	None	none (*in vitro*)	None	antibacterial	Bone regeneration scaffolds (*E. coli*, *S. aureus*)	Released Ga:∼0.03 mg/mL	None	2020	Li et al.^121^
	Ga-Ti-Al-Zr-Si	None	none (*in vitro*)	None	antibacterial	Dental/Orthopedic implants (*MRSA*)	Ga: 1, 2, 20, and 23 *wt* %	None	2019	Cochis et al.^122^
	Ga	None	interventional local drug administration	∼100 μm	X-ray images	Pig hearts and kidneys	4728∼5910 mg	None	2014	Wang et al.^33^
	LM/CA hydrogel	CA Hydrogels	interventional local drug administration	None	CT imaging; antitumor	Transcatheter arterial chemoembolization	Ga: ∼1573 mg/mL	Ga: ∼52 mg/kg	2019	Fan et al.^123^
	Fe@EGaIn/CA	CA (embolization and drug-loading); DOX·HCl (antitumor)	interventional local drug administration	∼500 μm	CT imaging and MRI; antitumor	Transcatheter arterial chemoembolization (VX2 cancer cells)	0∼200 mg/mL	Ga: ∼3 mg/kg	2021	Wang et al.^34^
	Liquid metal microspheres	Mineral oil containing surfactant (span80, 2 wt %)	intratumoral injection	∼300 μm	antitumor	magnetic thermotherapy	None	Ga: ∼11797 mg/kg	2023	Yang et al.^51^
			interventional local drug administration	∼300 μm	antitumor	transcatheter arterial embolization	None	Ga: ∼100 mg/kg		
	GMs	Chitosan	intratumor injection	∼200 μm	CT imaging and MRI; antitumor	tumor cryoablation (C8161 tumor cells)	None	Ga: ∼11820 mg/kg	2020	Sun et al.^35^
	DSPE-PEG2000-Amine-DC(8,9)PC-LM	DSPE-PEG2000-Amine (Surfactant); DC(8,9)PC (Surfactant)	intratumor injection	∼150 nm	PA imaging; antitumor	EGFR-positive human colon adenocarcinoma (HT29 cells)	25∼1600 μg/mL	Ga: ∼3624 mg/kg	2017	Chechetka et al.^24^
Gallium-based LMs biomedical devices	GaNP/Si	Si wafers (depositing Ga)	none (*in vitro*)	<50 nm	DNA sensing	peripheral blood leukocytes from cystic fibrosis patients	None	None	2016	Marin et al.^37^
	Oxidized GaIn	None	epidermal coating	none	magnetic induction heating	physical therapy and drug delivery (C8161 tumor cells)	Ga: ∼47.2 mg	Ga: ∼23593.8 mg/kg	2019	Wang et al.^49^
	MPC-PLC	poly(l-lactide-co-ε-caprolactone) (substrate)	implantation (Invasive)	LM particles: 2 μm Membrane: Thickness 50 μm, Length 2 cm, Diameter 2 mm	electrical stimulation electroporation	cardiovascular diseases (Electronic blood vessel)	Single device: non-cytotoxic for HUVECs, SMCs, and HAFs	None	2020	Cheng et al.^124^
	saTPW	poly(l-lactide-co-ε-caprolactone) (substrate)	implantation (Invasive)	LM particles: 2.5 μm Wire: Width 0.5 mm, Length 80 mm	electrical stimulation	postoperative arrhythmias (temporary epicardial pacing wire)	Single device: non-cytotoxic for HAFs and HUVECs	Ga: ∼0.67 mg/kg	2021	Hang et al.^125^
	EGaIn/Pt	parylene C encapsulation Cr/Au/Pt electrodes Semiconductor channels SiO_2_ gate dielectric ITO gate electrodes Polyimide substrate	implantation (minimally invasive)	microelectrodes: height, 60 μm; diameter, 20 μm	electrical stimulation	vision restoration (Implantable ultrathin retina)	Single device: non-cytotoxic for human retinal pigment epithelium cells	None	2024	Chung et al.^126^
	Gallium-based MPC	1- decanol (Surfactant) Novobiocin sodium and ciprofloxacin (antibiotic) Cyclophosphamide (antineoplastic)	epidermal or subcutaneous Implantation	Ga nanoparticles ∼500 nm Drug-loaded electrode ∼4 cm	antibacterial; antitumor	smart drug delivery	Single device: non-cytotoxic for HUVECs	Released Ga:∼45 mg/kg	2025	Qi et al.^127^

Reproduced from Wang et al.^9^ under the terms of the Creative Commons Attribution License, https://creativecommons.org/licenses/by/4.0/.

Note: ICG, *indocyanine green*; *ESBL E. coli, extended spectrum beta lactamase Escherichia coli*, CP, *capsular polysaccharide*; Ta, *Tannin*; *E. coli*, *Escherichia coli*; *S. aureus*, *Staphylococcus aureus*; *S. Intermedius*, *Streptococcus. Intermedius*; *P. intermedia*, *Prevotella intermedia;* LMNDs, *liquid metal nanodroplets*; DOX, *Doxorubicin*; LM, *liquid metal*; EGCG, *Epigallocatechin gallate*; DSPE-PEG2000-Mal, *1,2-Distearoyl-snglycero-3-phosphoethanolamine-N-[maleimide(polyethylene glycol)-2000]*; PEG, *Polyethylene Glycol*; GOX, *glucose oxidase*; MSN, *mesoporous silica nanoparticle*; HA, *Hyaluronic acid*; GLM, *Galinstan-based liquid-metal*; IL, *Ionic liquid*; SNPs, *supernanoparticles*; MW, *microwave*; Ga/M/PPs, *Membrane coated Ga particles with paclitaxel*; MRSA*, multidrug resistant Staphylococcus aureus*; MBG, *mesoporous bioactive glass*; CHT, *chitosan composite scaffolds*; FAPI, *Fibroblast activation protein inhibitors*; CT, *computed tomography*; PET, *positron emission tomography*; CA, *calcium alginate*; MRI, *magnetic resonance imaging*; GMs, *Gallium microparticles*; CS, *Chitosan*; DSPE-PEG2000-Amine, *1,2-distearoyl-sn-glycero-3-phosphoethanolamine-N-[amino(polyethylene glycol)-2,000]*; DC(8,9)PC, *1,2-bis(10,12-tricosadiynoyl)-sn-glycero-3-phosphocholine*; PA, *photoacoustic*; EGFR, *epidermal growth factor receptor*; MDR, *multidrug resistance*; Tf, *Tfansferrin*; LMGNs, *leukocyte membrane-coated gallium nanoswimmer*; tNPs, *transformable liquid-metal nanoparticles*; GQDs, *graphene quantum dots*; saTPW*, Soft and absorbable temporary epicardial pacing wire;* HAFs*, Human aortic fibroblast cells;* HUVECs, *Human umbilical vein endothelial cells;* SMCs*, Human aortic smooth muscle cells;* MPC*, Metal-polymer conductor;* PLC, *Poly(l-lactide-co-ε-caprolactone).*

#### Nano-sized formulations

The construction of nano-sized formulations (e.g., nanomedicines, nano-sensitizers, and drug carriers) has emerged as an effective approach to improve therapeutic efficacy and reduce *in vivo* toxicity.^9^ These nano-sized formulations can enhance therapeutic efficacy by targeting and accumulating within the lesion sites, thereby increasing drug concentrations and prolonging the therapeutic duration. Meanwhile, optimized metabolic pathways, minimized interactions with normal tissues, and reduced local irritation may also contribute to reducing renal and systemic toxicity. Compared to the nanomedicines self-assembled by Ga(III) with ligands (Figure 2A),^16^^,^^17^^,^^18^^,^^95^^,^^128^ the nano-sized formulations derived from gallium-based LMs may offer new approaches to simplify therapeutic strategies and enhance the efficacy of targeted therapy (Figures 2B and 2C).^15^^,^^19^^,^^20^^,^^129^ As innovative formulations, the nano-sized gallium-based LMs not only serve as reservoirs to release the active pharmaceutical ingredient (Ga(III)), but also exhibit intrinsic therapeutic activities (e.g., electrochemical substitution, stimulus responsiveness, and catalytic performance).^9^^,^^19^^,^^39^^,^^55^^,^^57^^,^^129^ Despite these advantages, the toxicological effects of nano-sized gallium-based LMs remain to be cautiously handled. Basically, the structural (e.g., microgels, capsules, core-shell and heterogeneous systems, and so forth) and compositional (e.g., alloy composition, doping substances and additional surface modification, and so forth) diversity of these nano-sized gallium-based LMs complicates the accurate assessment of their toxicological effects.^2^^,^^9^^,^^14^ In such cases, it is difficult to determine whether the toxicity assessment should focus on the “materials” themselves or their “degradation products”. More critically, the existing toxicity assessments often deviate from actual *in vivo* conditions and fail to accurately reflect the substance’s toxicity. For example, in short-term cell co-incubation assays that usually last less than 72 h,^15^^,^^19^^,^^20^ incomplete release of Ga(III) ions due to the surface oxide layer and modifications may misleadingly suggest that these nano-sized gallium-based LMs are low-toxicity or non-toxic. As early as 2018, Hyung-Jun Koo’s group demonstrated that gallium-indium eutectic alloy (EGaIn) is reasonably safe to use in an aqueous environment; However, caution should be exercised when external excitations (e.g., mechanical agitation) are applied, which sharply accelerates metallic ion leaching, especially In ions.^40^ Nevertheless, conclusions regarding their *in vivo* safety are generally considered reliable, since the doses of these nano-sized gallium-based LMs are typically within safe limits (<5 mg/kg, below LD_50_, Table 2).

#### Bioactive materials

As macroscopic-scaled formulations, gallium-containing bioactive materials that are typically administered via implantation or transdermal routes also warrant special attention (Table 2). The incorporation of gallium (Ga(0) or Ga(III)) as dopants in various bioactive materials (Figure 2D), including bioactive glasses,^97^ bioceramics,^130^ hydrogel,^131^ scaffolds,^132^ fibers,^133^ and composites,^134^ may offer additional therapeutic activities for dental/orthopedic repair, wound healing, and other biomedical applications (Table 2). In particular, the bioactive glasses or bioceramics that serve as postoperative regeneration scaffolds may enable the precise delivery of Ga(III) ions to the desired site, thereby enhancing therapeutic efficacy, including antibacterial, anti-inflammatory, antitumor, and osteogenic effects.^96^^,^^97^^,^^99^^,^^130^^,^^135^ However, the toxicity of these bioactive materials is highly correlated with the gallium dose. For example, excessive gallium addition (up to 7 mol %) within bioactive glass may increase the release of Ga(III) ions, leading to significant cytotoxicity.^45^^,^^136^ In addition to the release of Ga(III) ions, the *in vivo* interfacial stability and safety of gallium-based LMs also raise safety concerns. Under physiological conditions, the formation of oxide/oxyhydroxide (e.g., Ga_2_O_3_, GaOOH) through interfacial reactions will not only regulate Ga^3+^ release but also modulate their mechanical and biological interfacial properties.^137^ These oxide/oxyhydroxide may significantly affect the morphology, surface roughness, and adhesion performance, which further induce persistent tissue irritation and local inflammation. Early research has indicated that the LD_50_ values of gallium alloy (Ga: 65.4 wt %, In: 21.4 wt %, Cu: 9.6 wt %, Sn: 1.4 wt %, Zn: 2.2 wt %) used as a dental filling material, was 580 mg/kg (i.e., Ga = 379.3 mg/kg) for male rats, 493 mg/kg (i.e., Ga = 322.4 mg/kg) for female rats, 377 mg/kg (i.e., Ga = 246.6 mg/kg) for male mice, and 430 mg/kg (i.e., Ga = 281.2 mg/kg) for female mice.^138^ However, the gallium-based LMs that are utilized as structural substrates typically require high doses to exert specific therapeutic effects. For example, in the case of tumor arterial embolization, the dose of macroscopic gallium-based LMs utilized as liquid or microsphere embolic agents far exceeds the Ga LD_50_ values (by doses several dozen times.^51^ Therefore, the uncoated/unmodified macroscopic gallium-based LMs are unsuitable for long-term implant applications, especially for dental, orthopedic, and arterial embolization scenarios.

#### Biomedical devices

In recent years, gallium-based LMs have emerged as key materials for next-generation bioelectronics, which are widely used in wearable health monitoring, neural electrodes, tumor electrotherapy, and artificial organs, and so forth. (Figure 2E).^21^^,^^124^^,^^125^^,^^126^^,^^127^^,^^139^^,^^140^^,^^141^^,^^142^^,^^143^ To maintain optimal electrical conductivity, these biomedical devices usually require additional gallium-based LMs, which poses greater challenges to their long-term biosafety. Generally, flexible electronics,^144^ drug delivery patches^127^ for transdermal application can be considered non-toxic, mainly because the polymer encapsulation limits the release of Ga(III). However, improper packaging may lead to adverse effects, such as leakage, short circuits, and local irritation, which have not been adequately assessed. Although extensive reports have claimed that these biomedical devices are generally biodegradable and have low toxicity *in vivo*, there remain significant drawbacks. (1) The dosage is unclear: in most studies, the exact elemental content of gallium was not specified, with uncertainty regarding whether it falls within safe limits (<LD_50_).^125^ (2) Long-term *in vivo* safety remains unclear: similar to the bioactive materials mentioned above, the potential hazards associated with residual or degraded gallium components remain unclear, particularly for long-term implantation.

## Summary and future perspectives

Among diverse metallic materials suitable for biomedical translation, gallium-based liquid metals (LMs) have emerged as a highly promising category, due to their unique pharmacological activities and physicochemical properties. However, the release of Ga(III) ions from gallium-based LMs represents a double-edged sword and constitutes a central challenge to their safe and effective application. Under physiological conditions, the released Ga(III) ions act as redox-inert Fe(III) competitive inhibitors for broad-spectrum biomedical applications. Conversely, nonspecific biodistribution or unintended Ga(III) exposure may disrupt iron metabolism in healthy tissues, thereby triggering adverse downstream biological effects. Hence, the trade-off between efficacy and safety has become a key prerequisite for the clinical translation of gallium-based LMs. Although research in this field has grown rapidly, many biosafety concerns associated with gallium-based LMs have not yet been fully clarified, particularly regarding complex influencing factors. This review systematically summarizes the therapeutic activities and toxicological characteristics of gallium-based LMs. Herein, based on current analysis, the unresolved challenges and future perspectives for achieving an optimal balance between efficacy and safety are proposed to inspire innovative insights for further research.

### The unresolved challenges for toxicological research

The toxicity of gallium-based LMs primarily originates from their degradation products, mainly Ga(III) ions. Within pathological areas, Ga(III) ions may interfere with iron homeostasis via competitively binding to iron-binding proteins, thereby dysregulating various crucial cellular processes. Although the Ga(III) substitution holds promise for broad-spectrum therapeutic applications with a relatively low risk of inducing resistance, it also exerts side effects on normal cells. Since Ga(III) ions serve as both the therapeutic and toxic components, it remains challenging to accurately assess their toxicological profiles and implement rigorous safety control.

#### Challenges in isolating components

In toxicity assessments, it is hard to fully differentiate the intrinsic toxicity of free Ga(III) ions from that of bulk gallium-based LMs. In practical applications, the compositional diversity of the gallium-based LMs, such as GaIn_24.5_, GaIn_21.5_Sn_10_, and Galn_25_Sn_13_Zn_1_, complicates the separation of individual components during toxicological assessments.^2^ Additionally, the varied compositional modifications and structural alterations further exacerbate this predicament. Moreover, the intricate degradation products, such as ions (e.g., Ga^3+^, In^3+^, Sn^4+^), hydrolysis mixtures (e.g., Ga(OH)_11_^8−^, Ga(OH)_4_^-^), oxides (e.g., Ga_2_O_3_), and precipitates (e.g., GaOOH, Ga(OH)_3_), also warrant in-depth investigation. Given that toxicological research on single metallic elements has formed relatively systematic frameworks and produced conclusive results, future studies are advised to report the composition (e.g., Ga = XXX wt %, In = XXX wt %) and dosage (e.g., Ga = XXX mg/mL for *in vitro* dose, Ga = XXX mg/kg for *in vivo* dose) of gallium-based LMs following unified standards. Accordingly, subsequent research must guarantee that each individual component remains below its respective safety limit (e.g., < LD_50_), so as to establish a solid foundation for comprehensive biosafety assessment.

#### Complexities arising from dynamic changes

Following administration *in vivo*, the gallium-based LMs cannot remain statically stable, which also represents a double-edged sword. Instead, the gallium-based LMs may undergo dynamic changes (e.g., oxidation, dissolution, deformation, electrochemical reactions) in response to the physiological environment and external stimuli, which may confer additional therapeutic activity. Nevertheless, the continuous evolution of morphology, composition, and physicochemical features resulting from these dynamic behaviors may also trigger time-dependent toxicity. Consequently, conventional toxicological assessments appear insufficient for accurately evaluating their *in vivo* biosafety. For instance, the Ga(III) ions are released persistently over time; thus, the toxic effects cannot be evaluated merely by final observations. Once unpredictable off-target phenomena occur, the electrochemical reactions associated with Ga(III) ion release will disrupt ionic equilibrium and intracellular redox homeostasis. While substantial evidence indicates that gallium-based LMs are generally nontoxic or slightly toxic, the dynamic toxicological mechanisms and possible hazards have not yet been fully elucidated. Therefore, in-depth exploration of their dynamic toxicological characteristics in complex physiological environments represents a key hurdle to overcome for future clinical applications.

#### Long-term biosafety requires systematic exploration

The long-term biosafety issues of gallium-based LMs constitute a prominent bottleneck, mainly stemming from the opaqueness of *in vivo* distribution and metabolism kinetics. Distinct from conventional “static’’ biomaterials, the gallium-based LMs exhibit markedly disparate cellular uptake, *in vivo* distribution, and metabolic routes, all of which pose greater challenges for systematic evaluation. For example, due to the dynamic behavior of gallium-based LMs, their precise localization within the body poses a considerable challenge. Besides, it remains unclear whether gallium-based LMs could be completely excreted through renal, hepatic, or intestinal routes, and so forth, or instead accumulate as persistent deposits that induce long-term local adverse responses (e.g., chronic inflammation, tissue damage, or even irreversible organ dysfunction). These uncertainties directly raise apprehensions about the chronic risks originating from residual/non-degraded components. Thus, future research should pay greater emphasis on the potential hazards induced by persistent gallium exposure, especially off-target biodistribution, and subsequent toxic manifestations (e.g., cellular damage, secondary genetic modulation/mutation and immune dysregulation).

### Future outlooks to balance efficacy and safety for representative formulations

To address the aforementioned challenges and achieve coordination between efficacy and safety, typical strategies and future research directions are proposed based on representative formulations.

#### Nano-sized formulations

The nano-sized gallium-based LMs can achieve multi-modal therapeutic effects, relying on their unique physicochemical properties and Ga(III) ion release behavior. While different strategies are required for the spatiotemporal regulation of efficacy and toxicity, considering distinct therapeutic mechanisms. For therapeutic systems primarily driven by stimulus-responsive physicochemical activities, such as photothermal effect, ROS generation, and catalytic reactions, surface functional modification and structural stabilization strategies need to be further optimized. For example, surface modification, including ligand assembly, polymerization, bioconjugation, and galvanic replacement, represents a feasible strategy to improve functionality and delay premature Ga(III) ion release during circulation. Nevertheless, as the degradation process progresses, Ga(III) ions will eventually be released, interfering with normal cells and generating off-target toxicological effects. Therefore, current nano-sized gallium-based LMs are only suitable for local injection into the lesion site (e.g., intratumoral injection). To retain therapeutic performance while minimizing unintended toxic effects caused by early Ga(III) ion leakage, there remains a persistent need for refined targeting strategies and activation approaches. For iron-blocking therapies that rely on Ga(III) ion release, research priorities should focus on reducing premature hydrolysis and non-specific release *in vivo*, thereby improving bioavailability and reducing nonspecific toxicity. For example, the nanomedicines constructed using polyphenol (e.g., quercetin) and gallium-based LMs may not only adhere to lesion sites (e.g., inflammatory bowel disease and infectious wounds), but also capture dissociated Ga(III) ions to form metal-polyphenol complexes, thereby improving bioavailability, amplifying therapeutic effect, and enhancing biocompatibility. These nanomedicines are adaptable to oral and topical administration routes, yet intravenous delivery is hampered by intrinsic trade-offs between therapeutic efficacy and systemic toxicity (e.g., intratumoral accumulation <0.7%, even when functionalized with tumor-specific antibodies).

#### Bioactive materials

Gallium-based bioactive materials, including embolic composites, dental/orthopedic implants, bone regeneration scaffolds, wound dressings and topical ointments, and so forth, are generally characterized by localized therapeutic action and relatively limited systemic exposure. Their safety optimization should be implemented based on the specific application scenario and gallium loading content. For long-term implanted biomaterials with high gallium loading, both the cumulative release concentration and release kinetics of Ga(III) ions must be quantitatively controlled to strictly confine ion exposure within therapeutic and safe thresholds. For example, gallium-based LMs primarily serve as energy sensitizers and imaging agents in embolic composites (e.g., microspheres, gels, and liquid formulations), thus excessive dissociation of gallium should be appropriately suppressed to avoid additional toxicity risks. As pivotal biomaterials for interventional therapy, current research emphasizes the degradable superiority of gallium-based LMs in these embolic composites, which enable postoperative vascular recanalization without additional surgery. However, such inherent degradability inevitably triggers continuous Ga(III) ion release, which conflicts with the goal of suppressing excessive Ga(III) ion leakage. In contrast, for therapeutic and implantable biomaterials, such as dental/orthopedic implants, bone regeneration scaffolds, and wound dressings, the localized and controllable Ga(III) ion release is essential to maintain effective antibacterial and anti-inflammatory effects. Thus, the localized concentration of Ga(III) ions must be maintained within the effective therapeutic range, while treatment should be discontinued if plasma concentrations exceed the permissible range. For transdermal biomaterials, including wound dressings and topical ointments, their safety is more manageable since the concentration of Ga(III) ions entering the bloodstream is relatively lower. Nevertheless, standardized post-use cleaning procedures are necessary to remove residual gallium-based LMs, thereby preventing prolonged local accumulation and potential hazards.

#### Biomedical devices

For gallium-based LMs in biomedical devices, including bioelectronics, flexible therapeutic patches, and drug delivery devices, the most effective strategy for striking a balance between efficacy and safety is proper encapsulation. These biomedical devices usually achieve therapeutic/diagnostic effects by leveraging the intrinsic physicochemical properties of gallium-based LMs, such as electrical conductivity for biological signal detection, electromagnetic responses for magnetothermal therapy, electrochemical corrosion enabling controlled drug release, and so forth. In most cases, polymer encapsulation is a standard and necessary practice, as it benefits structural stability and enhances functionality. However, continuous mechanical deformation, thermal effects, and electrochemical reactions during operation may lead to leakage of gallium-based LMs and unnecessary release of Ga(III) ions. Meanwhile, adverse effects such as non-targeted irritation, chronic inflammation, and tissue damage should also be thoroughly evaluated. The gallium-based LMs biomedical devices designed for transdermal application are usually considered relatively safe, while trace Ga(III) ions leaching even provide additional beneficial effects (e.g., antimicrobial). In contrast, for long-term implant applications, superior fabrication techniques (e.g., 3D printing, direct-writing, micro/nano manufacturing) are needed to achieve optimal electrical conductivity while reducing the addition of gallium-based LMs. Furthermore, improving interfacial adhesion, regulating surface tension, and optimizing multilayer encapsulation architectures will be essential for ensuring long-term device integrity and preventing material leakage.

In conclusion, although the toxicological mechanisms and long-term biosafety profiles of gallium-based liquid metals remain insufficiently understood, their clinical translation appears highly promising. With continued efforts toward systematic toxicological evaluation, rational material design, and precise control of the efficacy-safety balance, gallium-based LMs are expected to emerge as a new generation of multifunctional metallic biomaterials for diverse biomedical applications.

## Acknowledgments

This work was financially supported by the talented program of 10.13039/501100003459Guizhou University (No. X2023152), Guizhou University Basic Research Project (No. guidajichu [2024] 40), Guizhou Provincial Basic Research Program (Natural Science) (No. MS [2025] 687, No. MS [2025] 688, No. QKHJC [2024] Youth 292, and No. MS [2024] 516), and the 10.13039/501100001809National Natural Science Foundation of China (No. 82473819, No. 52606079, and No. 32660264).

## Author contributions

Conceptualization, D.W.; methodology, D.W. and J.L.; investigation, D.W., and S.H.; writing – original draft, D.W. and S.H.; writing – review and editing, D.W., Y.W., J.L., and W.P.; funding acquisition, D.W., Y.W., J.L., and W.P.; resources, D.W. and J.L.; supervision, D.W., J.L., and W.P.

## Declaration of interests

The authors declare no competing interests.
